# Undifferentiated carcinoma of the pituitary gland: A case report and review of the literature

**DOI:** 10.3892/ol.2014.1796

**Published:** 2014-01-14

**Authors:** HSUN-HWA LEE, SHIH-HAN HUNG, TE-MING TSENG, YUN-HO LIN, JU-CHUAN CHENG

**Affiliations:** 1 Department of Neurology, Taipei Medical University-Shuang Ho Hospital, Taipei, Taiwan, R.O.C.; 2 Department of Otolaryngology, Taipei Medical University Hospital, Taipei, Taiwan, R.O.C.; 3 Department of Otolaryngology, School of Medicine, Taipei Medical University, Taipei, Taiwan, R.O.C.; 4 Department of Pathology, Taipei Medical University Hospital, Taipei, Taiwan, R.O.C.; 5 Department of Ophthalmology, Taipei Medical University Wan Fang Hospital, Taipei, Taiwan, R.O.C.

**Keywords:** pituitary gland, cancer, undifferentiated carcinoma, irradiation, radiotherapy

## Abstract

Primary pituitary gland cancer is extremely rare. The current study presents the case of a patient diagnosed with pituitary cancer three months after completing surgery and post-operative chemoradiotherapy for hypopharyngeal cancer. In this report we discuss 57-year-old patient who presented with diplopia and ptosis four months following the completion of treatment for hypopharyngeal cancer. A poorly-differentiated pituitary carcinoma was located. Despite aggressive treatment and surgical excision with postoperative chemoradiotherapy, the disease progressed rapidly and the patient succumbed due to multiple metastases and organ failure. This case report indicates a possible correlation between irradiation and the development of pituitary cancer.

## Introduction

Primary pituitary gland cancers are rarely observed, and when they occur, they usually arise from pre-existing, secreting, invasive macro-adenoma (1). Adrenocorticotropic hormone (ACTH)- and prolactin (PRL)-secreting tumours are the most common (1). Overall, <140 pituitary carcinomas have been reported in the related English literature. The undifferentiated type of pituitary carcinomas are reported even less. Thus, despite the study by Furth *et al* which implied that there was correlation between radiotherapy and pituitary tumors, the true correlation between radiotherapy and pituitary cancer remains undetermined (2). The present study describes the case of a patient diagnosed with pituitary cancer four months after completing surgery and post-operative chemoradiotherapy for hypopharyngeal cancer.

## Case report

**Figure 1:**
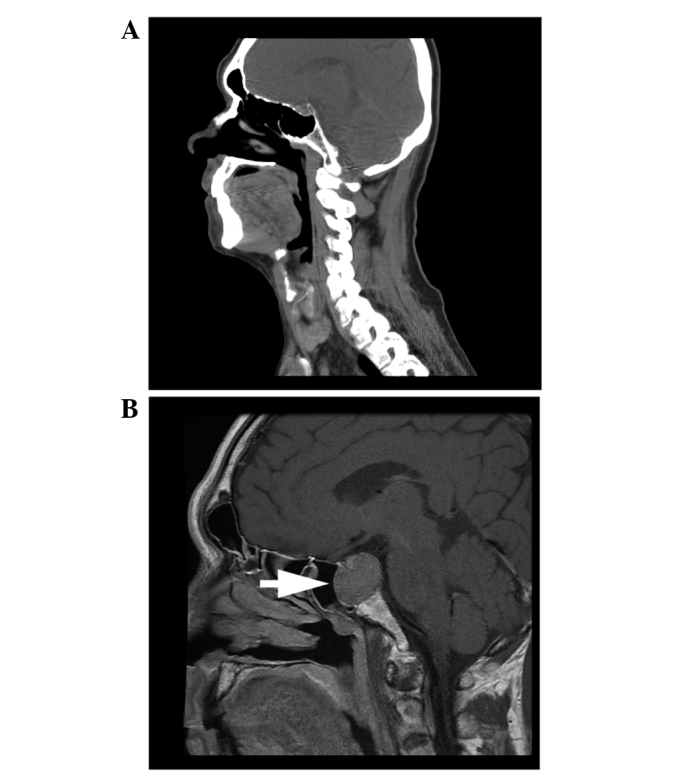
Sagittal view on T2-weighted magnetic resonance imaging at (A) two and (B) four months after completing treatment for hypopharyngeal carcinoma. A well-defined tumour in the pituitary fossa was revealed in subsequent imaging (arrow).

A 57-year-old male was consulted at Taipei Medical University Hospital, Taipei, Taiwan) for initial presentations of odynophagia and dysphagia. The patient was later diagnosed with stage IV hypopharyngeal squamous cell carcinoma. The patient underwent a laryngopharyngectomy with left radical neck dissection followed by post-operative concurrent chemoradiotherapy with cisplatin 3.85 mg/kg (total dose 250 mg) and irradiation of 6,600 cGy in 33 fractions. Imaging studies prior to and during treatment showed no abnormalities in the skull base region. However, four months after completing the treatment, the patient developed the symptoms of double vision and ptosis within a few days. Upon examination, there was limited medial motion of the left eye. Subsequent magnetic resonance imaging (MRI) revealed a well-defined mass at the pituitary fossa that partially obliterated the posterior lobe (Fig. 1). Surgical removal of the tumour was performed through a transnasal endoscopic approach.

**Figure 2:**
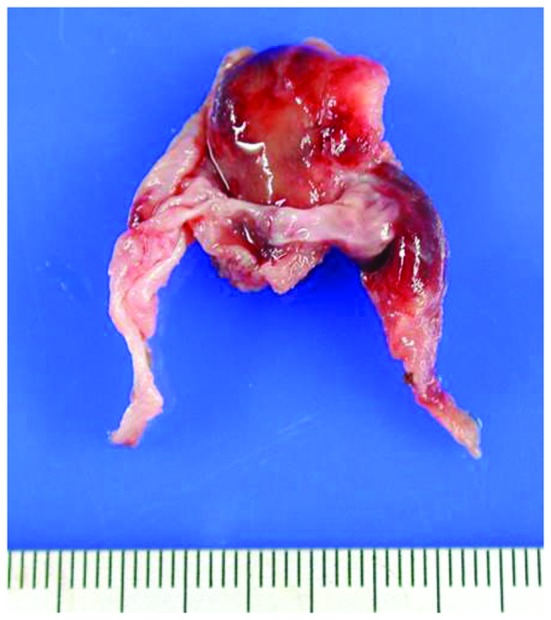
Gross appearance of the surgical specimen. Grossly, the tumour appeared brown and soft.

**Figure 3:**
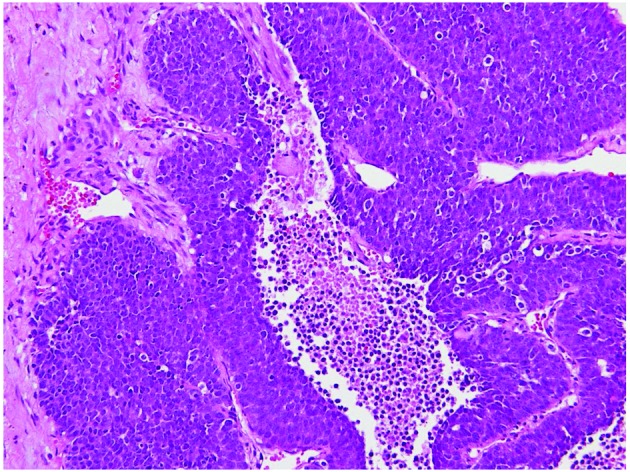
Microscopically, the tumour contained small round tumour cells in a solid sheet pattern, with focal tumour necrosis (H&E; magnification, ×200).

**Figure 4:**
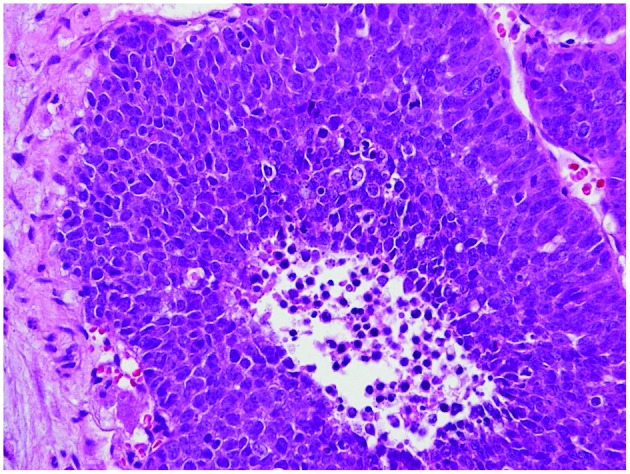
Tumour cells revealed a high nucleo-cytoplasmic ratio, hyper-chromatic nuclei, occasional nucleoli and frequent mitoses (H&E; magnification, ×400).

**Figure 5:**
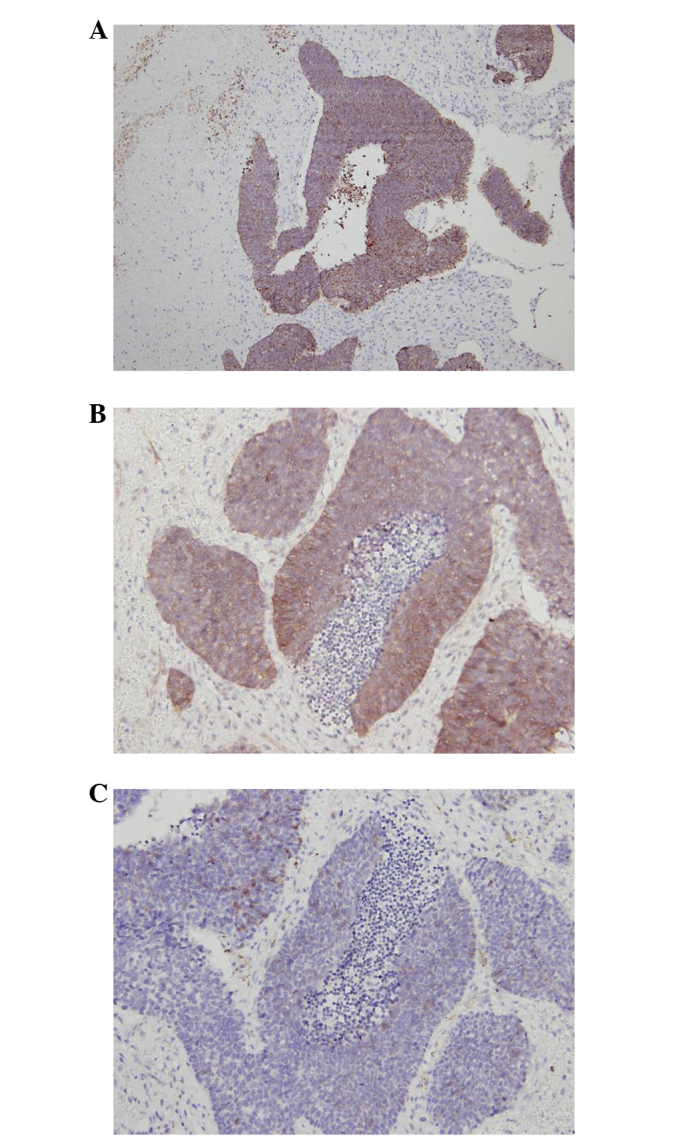
By immunohistochemistry, the tumour cells were diffusely weak to moderately positive for cytokeratin (A), diffusely positive for cluster of differentiation (CD)117 (magnification, ×100), (B) focally positive for CD56 (magnification, ×200) and (C) negative for cytokeratin (CK)7, CK20, chromogranin, synaptophysin, CK5/6, p63, S-100 and CD99 (magnification, ×200).

Grossly, the tumour appeared brown and soft (Fig. 2). Microscopically, it contained small blue round tumour cells in a solid sheet pattern, with focal tumour necrosis (Fig. 3). Focal haemorrhage and multi-nucleated giant cell formation within the stroma were also observed. The tumour cells revealed a high nucleo-cytoplasmic ratio, hyperchromatic nuclei, occasional nucleoli and frequent mitoses (Fig. 4). As observed using immunohistochemistry, the tumour cells were diffusely weak to moderately positive for cytokeratin, diffusely positive for cluster of differentiation (CD)117, focally positive for CD56 and negative for cytokeratin (CK)7, CK20, chromogranin, synaptophysin, CK5/6, p63, S-100 and CD99 (Fig. 5). Based on the morphology and immunoprofile, the diagnosis was of a poorly-differentiated carcinoma with focal neuroendocrine differentiation.

Following resection of the pituitary tumour, the patient received six cycles of chemotherapy with dacarbazine, epirubicin and cisplatin, and adjuvant irradiation to the skull base of up to 5,000 cGy in 25 fractions. The tumour did not appear to be responsive to the treatment. Primary tumour progression was noted during the post-operative treatment, and the patient developed bilateral vocal fold paralysis and intermittent, but massive, nasal bleeding. Whole spine MRI revealed several bone metastases complicated by pathological compression fractures at the T4–T5 level, with spinal cord compression. The patient’s general condition continued to deteriorate until he succumbed due to multiple organ failure. Written informed consent was obtained from the patient’s family.

## Discussion

In total, ~10–20% of the general population may have pituitary tumours (3). Of all pituitary tumours, the most common type is a pituitary adenoma. The diagnosis of benign, non-invasive pituitary tumours also includes hyperplasia, adenomas, cranio-pharyngiomas, meningiomas and pituicytomas. The prevalence of benign pituitary adenomas is ~16.7% (4). Pituitary malignancies include primary and metastatic cancers. Pituitary carcinomas are extremely rare, accounting for only 0.1–0.2% of all pituitary tumours (5). Common sites of metastasis include local areas such as the brain, spinal cord, lepto-meninges and cervical lymph nodes, or systemic areas, such as the liver, ovaries and bone (5). The majority of metastatic pituitary carcinomas originate from the breast and lungs, although other origins include the thyroid, prostate and salivary gland (6).

Pituitary tumours, both benign and malignant, are usually found with the following clinical observations: i) Oversecretion of certain hormones; and ii) subsequent ‘mass effects’ causing visual field impairment, headaches, diplopia or other neurological deficits (7). However, diagnoses occasionally turn out to be based on an incidental finding, with no clinical signs or symptoms.

Similar to benign pituitary adenomas, pituitary carcinomas are classified into hormone-secreting, invasive macro-adenomas and non-secreting carcinomas. Non-secreting pituitary carcinomas usually result in symptoms caused by mass effect. Among the secreting pituitary carcinomas, 42% produce ACTH while others produce PRL, growth hormone (GH), luteinizing hormone, follicle-stimulating hormone and thyroid-stimulating hormone (1). Histological features for primary pituitary carcinomas are significant nuclear pleomorphism and/or hyper-chromasia with increased proliferative activity, cytological activity and mitosis (5). Overexpression of the tumour suppressor oncoproteins p53 and Ki-67 is noted in pituitary carcinomas and adenomas (8). Compared with pituitary adenomas, pituitary carcinomas present with increased apoptosis, cyclooxygenase-2 expression and hypoxia-inducible factor-1α expression, and lower B-cell lymphoma 2 (anti-apoptotic factor) and p27 KIP1 levels (9).

Treatment options include tumour resection, hormone therapy, radiotherapy and chemotherapy. Surgical resection may not prolong survival, but it is important for providing the immediate relief of symptoms, particularly when the mass effect is a major concern. Surgery also aids in the accurate establishment of the pathological diagnosis. Nonetheless, early recurrence with rapid local growth, even after initial pituitary tumour resection, is common. Radiotherapy may be considered for additional local tumour control (3).

Hormone therapy with dopamine agonists (for PRL-producing tumours) and somatostatin analogs (for GH-producing tumours) are used to control the biochemical secretions. The chemotherapy commonly used for pituitary carcinomas is cyclohexyl-chloroethyl-nitrosourea, combined with 5-fluorouracil and/or temozolomide (3).

Pituitary carcinomas are generally associated with a poor prognosis. The mean survival time for patients is two years; however, the majority of patients succumb within one year of the discovery of the pituitary carcinoma (1,9).

Since the majority of pituitary malignancies arise from pre-existing benign pituitary lesions, the pathogenesis of the undifferentiated pituitary carcinoma of this case report is quite difficult to understand. In the post-treatment follow-up of the hypopharyngeal tumour, computed tomography revealed that two months prior to the diagnosis of pituitary cancer, the pituitary fossa appeared to be completely normal. Thus, there is suspicion of a link between irradiation and the pituitary cancer. Post-radiation sarcomas are already known as rare and long-term complications of radiotherapy (10); however, few studies discuss the topic of radiation-induced non-sarcomatous tumours (11,12).

In the present case, the radiation exposure of the pituitary gland during the treatment for hypopharyngeal cancer was relatively low. Moreover, there was only a three-month latency period for the pituitary cancer. Considering the differing nature of sarcomas and undifferentiated carcinomas, there be a yet unknown correlation between irradiation and the development of pituitary cancer in this patient. More studies are warranted to clarify this association. In the meantime, physicians should be more alert, as irradiation may cause more damage than previously expected.
